# Salivary Markers and Microbial Flora in Mouth Breathing Late Adolescents

**DOI:** 10.1155/2018/8687608

**Published:** 2018-03-05

**Authors:** Stefano Mummolo, Alessandro Nota, Silvia Caruso, Vincenzo Quinzi, Enrico Marchetti, Giuseppe Marzo

**Affiliations:** ^1^MeSVA, University of L'Aquila, L'Aquila, Italy; ^2^Dental School, Vita-Salute San Raffaele University, Milan, Italy

## Abstract

**Objective:**

This is a 6-month observational case-control study that aims to estimate plaque index (PI), salivary flow, buffering capacity of saliva, and specific* Streptococcus mutans (S. mutans)* and* Lactobacillus* rates in a mouth breathing late adolescents sample, after a professional oral hygiene procedure and home oral hygiene instructions.

**Subjects and Methods:**

A sample of 20 mouth breathing late adolescents/young adults (average: 19.2 ± 2.5; range: 18–23 years) and a matched control group of nose breathing subjects (average: 18.3 ± 3.2; range 18–23 years) were included in the study. All the participants were subjected to a professional oral hygiene procedure and appropriate home oral hygiene instructions (t0). After three months (t1) and six months (t2), the PI, salivary flow, buffering capacity of saliva, and* S. mutans* and* Lactobacilli* rates were recorded.

**Results:**

The mean buffering capacity of saliva and the salivary flow rate showed no significant difference between the two groups, all over the observational period. For PI, a significantly higher mode (score 1 of PI) was observed in the study group at t1 (score 0 = 35% of subjects; score 1 = 60%; score 2 = 5%) and t2 (score 1 = 65% of subjects, score 2 = 35%), with respect to control group. Furthermore, mouth breathing subjects show a significant 4 times higher risk to develop* S. mutans* CFU > 10^5^ (CI lower limit: 0.95; CI upper limit: 9.48; chi-square: 4.28; *p* = 0.03), with respect to the control subjects.

**Conclusions:**

Mouth breathing late adolescents show a significantly higher risk to develop* S. mutans* CFU > 10^5^ and an increased level of PI. Interceptive orthodontic treatments in growing subjects, like palatal expansion, are encouraged to improve the nasal air flow. In older subjects, orthodontic treatments should be performed with removable appliances like clear aligners, in order to allow a better oral hygiene level.

## 1. Background

Mouth breathing is breathing through the mouth rather than the nose. Generally speaking healthy humans may breathe through their nose, their mouth, or both. During rest, breathing through the nose is common for most individuals, while breathing through both nose and mouth during exercise is also normal. Mouth breathing may be called abnormal when an individual breathes through the mouth even during rest [1].

In about 85% of cases, mouth breathing represents an involuntary, subconscious adaptation to reduced patency of the nasal airway, and mouth breathing is required simply in order to get enough air [2, 3].

Chronic mouth breathing in children may have implications on dental and facial growth and also may cause gingivitis (inflamed gums), caries, and increased levels of dental plaque.

It appears that mouth receives a greatest exposure to airflow during breathing and, consequently, the gingival inflammation and irritation could be related to surface dehydration and reduced salivary flow [4, 5].

Also caries has been placed in correlation with the entry of air through the mouth (associated with the surface dehydration and disappearance of the film of saliva from the tooth surface); the regular intake of fermentable carbohydrates in the diet involves lowering the pH, resulting in the formation of plaque mostly composed of acid-forming and acid-tolerable species, such as* Streptococcus mutans (S. mutans)* and* Lactobacilli *[6–8]. The association with the colonies of* S. mutans* and* Lactobacilli* in saliva is present in children with allergic rhinitis [9] and also in young adults with asthma [2].

In mouth breathing adolescents a reduction of buffering capacity of saliva and its flow rate was also observed [1], concluding with a general belief about the necessity of prevention programs in mouth breathing individuals to maintain a proper oral health status. Literature, however, lacks data about any preventive programs in mouth breathing subjects and their impact on salivary parameters.

This 6-month observational case-control study aimed to estimate PI, salivary flow, buffering capacity of saliva, and specific* S. mutans* and* Lactobacillus* rates in mouth breathing late adolescents.

## 2. Subjects and Methods

This study was conducted on a study group of 20 mouth breathing late adolescents/young adults (9 females and 11 males; mean age: 19.2 ± 2.5; range: 18–23 years) and a matched control group for age and gender, observed for the same time period of six months, submitted to a professional oral hygiene procedure and home oral hygiene instructions. Subjects were recruited in the Dental Clinic of the University of L'Aquila, Italy, and the ethical committee or this institution approved the protocol of the study. Furthermore, each subject signed an informed consent form prior to the beginning of the study.

At baseline, prior to the enrolment, the subjects were interviewed and visited regarding their breathing environment. Other criteria were that the subjects should be free of systemic diseases, should not be taking any medication, and should not have teeth with active caries in their dental arches [1].

The mouth breathing ascertainment was made through medical history, clinical examination, and some clinical tests described in Table 1. A control group of 20 matched subjects, with nose breathing pattern (9 females and 11 males, mean age: 18.3 ± 3.2, range: 18–23 years), was also enrolled. In particular, for each mouth breather, an age-matched control subject was enrolled, with the closest gender proportion to each case, in a 1 : 1 proportion. The subjects were followed for a period of six months, and plaque index (PI) [10], salivary flow rate (sf), buffering capacity of saliva, and the* S. mutans* and* Lactobacilli* CFU counts were recorded for each subject at the beginning of the study (t0) and after three (t1) and six months (t2). The outcomes of the study are reported in Table 2. As the home oral hygiene habits could be* potential confounders,* a few days before the beginning of the observational period, a professional oral hygiene procedure was performed and accurate home oral hygiene instructions were given to each subject. At each appointment (at t0, t1, and t2), refraining from eating, drinking alcohol, smoking, or brushing the teeth for at least one hour prior to the visit has been recommended to patients, as all these actions could alter the average salivary flow. The samples of saliva were collected between 09:00 and 10:00 a.m. to reduce possible circadian interference. At each appointment, firstly, the PI was recorded. Then, the patient was administered a stimulant paraffin tablet that had to be chewed for 30 seconds, then inviting him/her to expel the saliva produced. These samples of saliva were subjected immediately to evaluation of buffering capacity. The buffering capacity of the saliva was assessed through the* CRT® buffer* (Ivoclar Vivadent Clinical, Schaan, Liechtenstein), which was expressed according to the manufacturer's instruction in five scores (Table 2). Subsequently, the subject was invited to chew it for 5 minutes, collecting all the saliva produced in a graduated glass. Through this procedure, the ml of saliva was collected and the salivary flow rate for minutes (ml/min) was calculated. Subsequently, a part of each saliva sample was used for bacterial count through the* CRT bacteria* (Ivoclar Vivadent Clinical, Schaan, Liechtenstein).

### 2.1. Bacterial Count

From the graduated glass, previously filled by the patient, another part of the saliva sample was used to completely wet the culture media (agar) using pipettes. A NaHCO_3_ tablet was inserted in the container of the culture agar to stimulate the growth of bacteria and they were placed in an incubator at 35–37°C for 48 hours. Then, the obtained results were evaluated.* S. mutans* colonies appeared as small blue colonies with a diameter < 1 mm on blue agar, while* Lactobacilli* colonies presented themselves as white colonies in transparent agar. The presence of a bacterial count higher than 10^5^ CFU/ml of saliva indicates a high risk of developing tooth decay. Thus, in this study, subjects were divided as* S. mutans* and* Lactobacilli* counts > or <10^5^ CFU/ml [11–14].

### 2.2. Statistical Analysis

Statistical analysis was performed using the SPSS software (SPSS Inc., Chicago, IL).

A Student's *t*-test was applied to confirm the absence of differences in age between the study group and the control group.

For the PI, an ordinal variable, the data are presented as the mode value and the number of subjects (in percentage over the sample) for each of the scores. Significant intragroup differences were calculated with the Friedman test, and Wilcoxon signed rank test was used as post hoc evaluation. Between-groups differences were calculated with the Mann–Whitney *U* test.

The same tests were used for the scores indicating the buffering capacity of saliva.

For the salivary flow rate, a continuous variable, the results are presented as mean and SD. Significant between-groups differences were determined by Student's *t*-test, and the ANOVA analysis was used to calculate intragroup differences.

For the* S. mutans *and* Lactobacilli *counts, the results are presented as the number of subjects (in percentage over the sample) with a CFU count > 10^5^. This method of measuring the oral microbiota was shown to be valid in a similar cohort study from adolescents [13]. The relative risk was calculated for* S. mutans* > 10^5^ and* Lactobacillus* > 10^5^ in the compared groups. The 95% confidence interval (CI) was computed around the adjusted relative risk, using the variance according to chi-square test.

The level of significance was set at *p* < 0.05.

## 3. Results

This 6-month observational case-control study analyzed plaque index (PI), salivary flow, buffering capacity of saliva, and specific* Streptococcus mutans* (*S. mutans*) and Lactobacillus rates in two age-matched (absence of statistically differences in age according to Student's *t*-test; *p* > 0.05) groups of late adolescents with and without mouth breathing habits.

The results are reported in Tables 3–6.

### 3.1. Salivary Flow Rate

No significant differences were observed in the salivary flow rate all over the observational period. The mean values were included in a range between 1.22 ml/min and 1.56 ml/min, for the two groups (Table 3).

### 3.2. Plaque Index

Recorded data of PI in the two groups are reported in Table 4. At t0, the PI was 0 in almost all the subjects of both the study group and the control group. However, at t1, the cases showed a significant increase of scores 1 or 2, and subjects with score 0 passed from 100% of the study sample (20 subjects over 20) at t0 to 35% of the study sample (7 subjects over 20) at t1.

And at t2, none of the subjects of the study group had score 0, as the mode was PI = 1 (65% of cases).

In synthesis, the mouth breathing subjects showed an increasing trend of PI over time, with respect to the control group. The statistical analysis shows a significantly higher PI in the study group with respect to the control group at t1 and t2.

### 3.3. Buffering Capacity of Saliva

Table 5 summarizes the data about the buffering capacity of saliva. No statistically significant differences were found between the study group and the control group, all over the observational period. Moreover, no changes of this parameter are appreciable in each group during the follow-up. The value remained always in a score 3 (average level), all over the observational period, in the whole sample.

### 3.4. *S. mutans* and* Lactobacilli* Count

Table 6 shows the percentage of patients with* S. mutans* > 10^5^ CFU and* Lactobacilli* > 10^5^ CFU and the comparison between the cases and the controls, all over the observational period.

The mouth breathing subjects showed a significant 4 times higher risk to develop* S. mutans* > 10^5^ CFU (CI lower limit: 0.95; CI upper limit: 9.48; chi-square: 4.28; *p* = 0.03), with respect to the control subjects. At t2, 45% of mouth breathing subjects (9 subjects over 20) had* S. mutans* > 10^5^ CFU, compared with only 15% in the control group (3 subjects over 20).

In addition, the mouth breathing subjects showed a tendency to develop a* Lactobacilli* colonization, although not statistically significant (a 3.58 times higher risk to develop* Lactobacilli* CFU > 10^5^, CI lower limit = 0.83; upper limit = 14.83, chi-square: 3.58, *p* = 0.058).

## 4. Discussion

This observational case-control study aimed to assess plaque index, salivary flow, buffering capacity of saliva, and specific* S. mutans* and* Lactobacilli* rates in a sample of mouth breathing subjects during six months after a professional oral hygiene procedure and home oral hygiene instructions.

Based on the results, the mouth breathing subjects seem to be predisposed to develop a higher PI compared with healthy subjects (although never higher than score 2). Moreover, the same subjects have a significant 4 times higher risk to develop* S. mutans* > 10^5^ CFU (CI lower limit: 0.95; CI upper limit: 9.48; chi-square: 4.28; *p* = 0.03), compared with healthy subjects. No differences were detected for the buffering capacity of saliva and for the salivary flow rate, between cases and controls.

To the best of the authors' knowledge, this is the first study in literature that provides reliable and controlled data, with a follow-up period of 6 months, on the bacterial colonization and the salivary parameters in a group of late adolescents with oral breathing. In addition, the present data are recorded after a professional oral hygiene procedure and proper oral hygiene instructions, to avoid the effect of the oral hygiene as* potential confounders. *

### 4.1. Plaque Index

At t0, the mode of PI was 0 in both the case and control groups. And no subjects showed PI > 1.

However, at t1, there is a significant increase in the cases of scores 1 or 2 in the study group, and subjects with score 0 decreased from 100% of the sample (20 subjects over 20) at t0 to 35% of the sample (7 subjects over 20) at t1. At t2, none of the subjects of the study group had score 0, as the mode was PI = 1 (65% of cases). The statistical analysis showed a significantly higher PI in the study group, with respect to the control group, at t1 and t2. Thus, oral breathers seem to be predisposed to a higher accumulation of dental plaque.

This result is in accordance with a previous case-control study performed on adults with asthma, in which a higher plaque score was found among the asthmatics subjects (mostly oral breathers) compared with control subjects [15].

Despite the significant differences between cases and controls, it is important to point out, from a clinical point of view, that no subjects—among the oral breathers, as well as the controls—have ever developed a PI > 2, all over the observational period. Even at t2 (6 months after professional oral hygiene procedure and home oral hygiene instruction), the cases showed a mode of PI = 1 (65% of cases), while PI = 2 was observed only in the other 35% of the cases. Control subjects never developed a PI = 2.

These data suggest that planning periodic (at least every six months) preventive professional treatments could help in maintaining an acceptable level of plaque index (PI = 0 or 1) in the majority of oral breathers late adolescents, despite their significant predisposition to develop higher PI with respect to healthy nose breathers.

### 4.2. Salivary Flow Rate

In this study no difference in the salivary flow rate was observed over time within groups or between groups. Considering that the normal value of salivary flow rate for an adult subject is ≥1 ml/min, while values lower than 0.7 ml/min are considered too low [16], our results suggest that mouth breathing late adolescents may present salivary flow rate levels, similar to healthy subjects.

In the previous literature, Koga-Ito et al. [16], in a case-control study conducted on 30 mouth breathing children and healthy subjects, found no difference in the salivary flow rate between the study and the control subjects. However, the study sample was composed of children and did not report a follow-up period or any preliminary professional oral hygiene procedure, so their results are not directly comparable with the present study.

In a sample of adolescents aged 10–19 years, Weiler et al. [1] compared the salivary flow rate and the buffering capacity of saliva in 30 mouth breathers and 31 control subjects and reported a salivary flow rate of 1.21 ± 0.53 ml/min in mouth breathers and 1.18 ± 0.71 ml/min in nose breathers, without significant differences between the two groups. Also in this case, the results are not directly comparable, due to the different age range and the absence of follow-up or preliminary dental procedure. In addition, Weiler et al. [1] discarded all the saliva produced in the first minute and a half from the beginning of stimulation, while we included this saliva in our calculation. This could partly explain the lower salivary flow rates reported by Weiler et al., with respect to the present data.

In addition, considering that the salivary flow rate seems to be influenced by the age [17], at this time, the “age factor” could probably also explain the different results among different studies.

More recently, Stensson et al. [2] reported the salivary flow rate of 20 young adults with asthma (that is associated with mouth breathing) and registered a statistically significant lower stimulated flow rate in the study population, respectively, 2 ± 0.6 ml/min in the study group and 2.8 ± 1.1 ml/min in the control subjects. Data from Stensson et al. [2] are not directly comparable with the present study, because they analyzed stimulated saliva after 3 days without brushing teeth and not always at the same time during the day. In addition, their data are not directly comparable with the present data because of the different sample (subjects with asthma).

Thus, the present data suggest that the salivary flow rate is not affected by mouth breathing. Consequently, in these patients, it is probably the airflow in the mouth that determines the dehydration of the mucosa and the surfaces of the teeth [5], even in the presence of a normal salivary flow rate. This problem (correlated to oral diseases) could be countered with the use of stimulants of saliva in mouth breathing subjects.

### 4.3. Buffering Capacity of Saliva

The present data report no statistically significant differences between the study and the control groups, all over the observational period, in the buffering capacity of saliva. For both the two groups, the buffering capacity remained at level 3 (average level) for the whole observational period, in all the subjects. This result indicates that oral breathing may not affect the buffering capacity of the saliva. This finding is also supported by other data in the previous literature, such as those from Weiler et al. [1], that reported values of 1.39 ± 0.24 in the oral breathing adolescents and 1.41 ± 0.34 in the control adolescent subjects, and from Koga-Ito et al. [16] who reported a similar buffering capacity of saliva in mouth breathers children, compared to healthy subjects. In addition, also in children with allergic rhinitis [9], buffering capacity of saliva was similar to that in control healthy subjects, assessed to a middle level of the manufacturer's test indications, suggesting that the respiratory function does not affect it.

The present data suggest that the buffering capacity of saliva tends to be normal and stable, even after a professional hygiene procedure and home oral hygiene instruction, in mouth breathers. Thus, procedures to increase the pH are not indicated in these cases.

### 4.4. Bacterial Count

In this study the amount of bacteria increased over the time in the two groups from t0 to t2. But mouth breathing subjects showed a significant 4 times higher risk to develop* S. mutans* >10^5^ CFU (CI lower limit: 0.95; CI upper limit: 9.48; chi-square: 4.28; *p* = 0.03), compared to the control subjects. At t2, 45% of mouth breathing subjects (9 subjects over 20), but only 15% in the control group (3 subjects over 20), showed* S. mutans* > 10^5^ CFU.

Table 3 also shows the percentage of subjects with* Lactobacilli *> 10^5^ CFU, and the comparison of the two groups and data suggest that only* S. mutans *colonies can significantly increase in mouth breathing subjects.

Previously in literature, it was demonstrated that mouth breathing children develop higher colonization by* S. mutans* compared with control healthy children, while no difference was observed in the* Lactobacilli* colonization [16].

Also, a study on children with allergic rhinitis reported no significant difference for the* Lactobacilli* colonies [9] (as* Lactobacilli* counts > 10^5^ were observed in 15% (6 over 40 subjects) both in case and control children) and reported that the allergic rhinitis is revealed as a risk factor for* S. mutans* colonization, as 57.5% (23 over 40 subjects) showed* S. mutans* > 10^5^, against 5% (2 over 40 subjects) in the healthy children.

Although based on different samples, these studies seem to confirm that respiratory function cannot influence* Lactobacilli* colonies in the mouth but influences* S. mutans* colonies in groups of children, adolescents, and young adults.

The data of the present study are constructed with a longitudinal design, and this allows the determination of a relative risk factor (risk to develop* S. mutans* > 10^5^ CFU; CI lower limit: 0.95; CI upper limit: 9.48; chi-square: 4.28; *p* = 0.03).

A possible explanation is that the group of mouth breathers may retain a greater amount of bacteria in their oral cavities, possibly due to the evaporation of water from the saliva of constant mouth breathers that can reach 0.24 ml/min [1, 18].

It is important to point out that the increase in* S. mutans* colonies occurred despite the oral hygiene procedure and instructions, as was the case in the plaque index. This suggests that standard treatments could be not effective in counteracting the mouth breathing risks for oral health. Adding salivary stimulants, even in cases with a normal salivary flow rate, in these patients could be useful in contrast to the salivary liquid evaporation. Similarly, frequent periodic examinations and oral hygiene professional treatment should be performed to help the maintenance of an appropriate oral hygiene status.

Furthermore, the reported increased presence of* S. mutans* and lower level of oral hygiene should be considered in the orthodontic treatment planning for mouth breathers subjects, where the increased difficulties in maintaining satisfactory levels of oral hygiene, combined with the use of fixed multibracket appliances [14], could determine an increased risk of iatrogenic effects such as dental caries or enamel demineralization, directly related to the level of oral hygiene and* S. mutans* colonies during the orthodontic treatment [19, 20]. Thus, in the orthodontic treatment of mouth breathing subjects, the use of removable appliances such as clear aligners, rather than fixed vestibular of lingual multibracket appliances, should be encouraged to counteract the individual predisposition to lower oral hygiene conditions [21–23].

For the same reasons and also to allow a better oral health level during the whole life, interceptive orthodontic therapies with a positive impact on the nasal air flow such as, principally, palatal expansion should be encouraged to improve the breathing pattern in growing subjects [24–27], while this is not possible in the age range of this study or later, where specific preventive protocols are more appropriate.

## 5. Conclusions

This study demonstrates that, although proper oral hygiene home instruction and professional hygiene procedures could be applied, mouth breathing late adolescents and young adults have a significant 4 times higher risk to develop* S. mutans* CFU > 10^5^ (CI lower limit: 0.95; CI upper limit: 9.48; chi-square: 4.28; *p* = 0.03), compared with the control subjects, and a significantly higher increase of plaque index in a period of 6 months. Thus, interceptive orthodontic treatments in growing subjects, such as palatal expansion, are encouraged in order to improve the nasal air flow. In older subjects, special oral health programmes with periodic visits (at least every six months) and other agents, for example, to increase saliva production, should be taken into consideration for mouth breathing late adolescents and young adults in order to maintain a good oral health. Orthodontic treatments should be performed with removable appliances such as clear aligners, in order to allow a better oral hygiene level and reduce the risk of development of iatrogenic effects such as white spots lesions or dental caries.

## Figures and Tables

**Table 1:** The primary item used for the ascertainment of mouth breathing.

Clinical examination	
Item 1	Nasal breathing individuals have their lips lightly touched during the relaxed breathing hours, whereas mouth breathers keep the lips apart.

Item 2	A normal nose breathing individual will usually dilate nostrils while taking a deep breath. A mouth breathing individual when asked to close the lips and take a deep breath will appreciably not change the shape and size of the external nares and may occasionally contract nasal orifices while taking a deep breath. This is because the nasal breathers may normally demonstrate a good reflex control of alar muscles that control the external nares shape and size. Even the nasal breathers with the temporary nasal congestion may demonstrate the dilation of the nares and reflex alar contraction during voluntary inspiration.

Clinical tests	

Mirror test	A cold mirror is placed under the subject's nostrils and he or she is asked to inhale and exhale through the nose. If moisture condenses on the mirror, this demonstrates that the patient has successfully exhaled through the nares.

Nares reflex	The nares reflex test shows whether or not the nose is functioning normally. While the subject's mouth is closed, the operator pinches the patient's nostrils for 2 seconds and then releases them: the alae of the nose should “flutter” in nasal breathers.

**Table 2:** Outcomes of the study.

Plaque index	For the PI index, the scores 0, 1, 2, 3 were reported

Salivary flow	The salivary flow was calculated as the amount of saliva (ml) produced in a given time frame (in this study 5 minutes). The salivary flow rate is the amount in a minute (ml/min).

Buffering capacity of saliva	The* CRT buffer* kit (Ivoclar Vivadent Clinical, Schaan, Liechtenstein) was used to calculate the buffering capacity of saliva, which was categorized in the following values: 1 = low; 2 = medium-low; 3 = average; 4 = medium-high; 5 = high.

*S. mutans* CFU count	The *CRT bacteria *kit (Ivoclar Vivadent Clinical, Schaan, Liechtenstein ) was used to assess the *S. mutans* colonies, which appeared as small blue colonies with a diameter < 1 mm on blue agar. Subjects were dichotomized as *S. mutans* count > or <10^5^.

Lactobacillus CFU count	The *CRT bacteria *kit (Ivoclar Vivadent Clinical, Schaan, Liechtenstein) was used to assess the *Lactobacilli* colonies, which appeared as white colonies with a diameter < 1 mm in transparent agar. Subjects were dichotomized as *Lactobacilli* count > or <10^5^.

**Table 3:** Distribution of data of salivary flow rate (ml/min) and statistical comparisons within and between groups.

	Salivary flow rate (t0)	t0 versus t1	Salivary flow rate (t1)	t1 versus t2	Salivary flow rate (t2)	t0 versus t2
Control group	1.22 ± 0.52	n.s.	1.25 ± 0.55	n.s.	1.56 ± 0.7	n.s.
Study group	1.27 ± 0.68	n.s.	1.52 ± 0.68	n.s.	1.23 ± 0.52	n.s.
Differences between groups	n.s.		n.s.		n.s.	

n.s.: not significant.

**Table 4:** Distribution of data of plaque index (PI) and statistical comparisons within and between groups.

	PI (t0)		t0 versus t1	PI (t1)		t1 versus t2	PI (t2)		t0 versus t2
Control group	0	85%	n.s.	0	100%	n.s.	0	90%	n.s.
	1	15%		1	0%		1	10%	
	2	0%		2	0%		2	0%	

Study group	0	100%	*p* = 0.000^*∗∗∗*^	0	35%	*p* = 0.005^*∗∗*^	0	0%	*p* = 0.000^*∗∗∗*^
	1	0%		1	60%		1	65%	
	2	0%		2	5%		2	35%	

Differences between groups		n.s.			*∗*			*∗*	

n.s.: not significant; ^*∗*^*p* < 0.05, ^*∗∗*^*p* < 0.01,^*∗∗∗*^*p* < 0.001.

**Table 5:** Distribution of data of buffering capacity of saliva (expressed as score and percentage of subjects in the whole sample) and statistical comparisons within and between groups.

	Buffering capacity of saliva (t0)		t0 versus t1	Buffering capacity of saliva (t1)		t1 versus t2	Buffering capacity of saliva (t2)		t0 versus t2
Control group	1	0%	n.s.	1	0%	n.s.	1	0%	n.s.
	2	0%		2	0%		2	0%	
	3	100%		3	100%		3	100%	
	4	0%		4	0%		4	0%	
	5	0%		5	0%		5	0%	

Study group	1	0%	n.s	1	0%	n.s.	1	0%	n.s.
	2	0%		2	0%		2	0%	
	3	100%		3	100%		3	100%	
	4	0%		4	0%		4	0%	
	5	0%		5	0%		5	0%	

Differences between groups		n.s.			n.s.			n.s.	

For the buffering capacity of saliva, 1 = low; 2 = medium-low; 3 = average; 4 = medium-high; 5 = high; n.s.: not significant.

**Table 6:** Distribution of data of bacterial count and statistical comparison**s** within and between groups.

		Subjects with CFU/ml > 10^5^ (t0)	t0 versus t1	Subjects with CFU/ml > 10^5^ (t1)	t1 versus t2	Subjects with CFU/ml > 10^5^ (t2)	t0 versus t2
S. Mutans	Control group	0%	n.s.	15%	n.s.	15%	n.s.
	Study group	0%	n.s	5%	*∗*	45%	*∗*
	Differences between groups	n.s.		*∗*		*∗*	

Lactobacilli	Control group	10%	n.s.	0%	n.s.	10%	n.s.
	Study group	0%	n.s.	5%	*∗*	35%	*∗*
	Differences between groups	*∗*		n.s.		*∗*	

n.s.: not significant; ^*∗*^*p* < 0.05.
